# Isolated antro-pyloric metastatic mass from colonic carcinoma: A rare case presentation

**DOI:** 10.1016/j.amsu.2018.11.004

**Published:** 2018-11-22

**Authors:** Samrat Ray, Amitabh Yadav, Sunila Jain, Samiran Nundy

**Affiliations:** a Surgical Gastroenterology and Liver Transplantation, Sir Ganga Ram Hospital, New Delhi, India; b Department of Histopathology, Sir Ganga Ram Hospital, India

**Keywords:** Metastatic, Gastric, Colonic, Adenocarcinoma

## Abstract

Metastatic tumors to stomach are extremely rare with very few cases being described so far in the surgical literature. Colonic tumors metastatic to stomach represent a rarer entity and present a surgical challenge for diagnosis and management to the clinician. We, hereby present a case of adenocarcinoma of transverse colon metastatic to stomach more than 6 years after the index malignancy, presenting clinically with features of gastric outlet obstruction. It was treated with open subtotal gastrectomy, with diagnosis being made on histopathologic examination using special immunochemical stains. Adjuvant treatment in the form of chemotherapy was given and follow up cross sectional imaging showed no evidence of residual disease so far.

## Highlights

• Extreme rarity of this pathological entity.
• Diagnostic challenges faced in such a clinical scenario.
• Forming a very rare and interesting differential diagnosis of gastric outlet obstruction.
• First case reported to present 6 years after the index colonic primary.

## 1 Introduction

Metastasis to the stomach is rare, with few cases reported in the literature. The most common sites of the primary malignancy have been found to be the lung, breast and skin (malignant melanoma) in a descending order [1] but colon cancer metastasising to the stomach is even rarer with probably only five cases reported [2]. To our knowledge, this is the first report of a metastasis from the colon to the stomach six years after surgery for the primary lesion. The work has been reported in line with the SCARE criteria [3].

## 2 Presentation of case

A 76-year-old lady, a known diabetic, hypertensive and hypothyroid presented with upper abdominal discomfort, bloating and repeated bouts of bilious vomiting for 5 days. Six years prior, she had undergone a right hemicolectomy for a caecal carcinoma. There was a 10 × 8 cm ulcero-proliferative mass in the caecum which was infiltrating posteriorly into the right psoas muscle. The omentum, peritoneum, mesentery and the other organs were free of any metastatic deposits. Histopathology showed a moderately differentiated adenocarcinoma of the caecum (pT3N0Mx); none of the 16 resected lymph nodes were involved and the proximal, distal and retroperitoneal margins were free of tumor. She underwent 6 cycles of Capecitabine based adjuvant chemotherapy and was being followed up as an outpatient. She was apparently well till 6 years ago, when she developed sudden upper abdominal discomfort associated with bilious vomiting. It was managed conservatively, and relevant investigations were done.

**Fig. 1:**
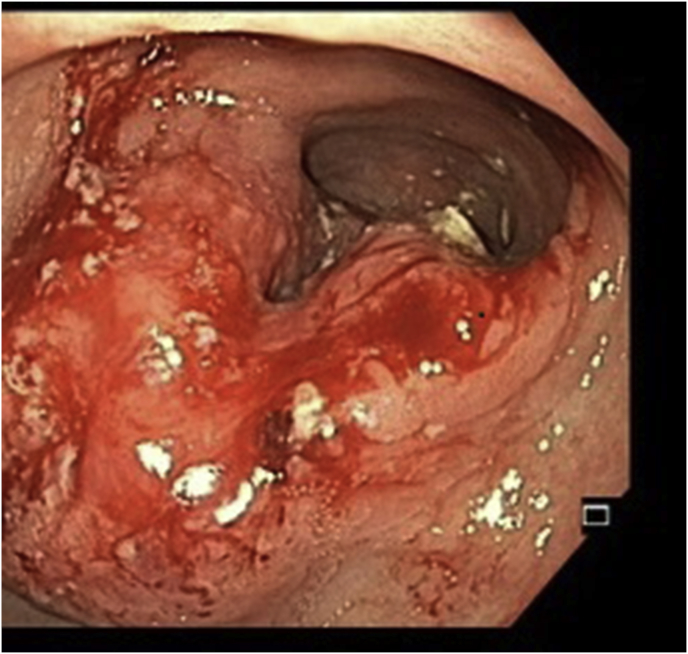
UGI endoscopy showing large ulcero-proliferative mass in the antro-pyloric region with normal overlying mucosa. Scope was not negotiated beyond the mass.

**Fig. 2:**
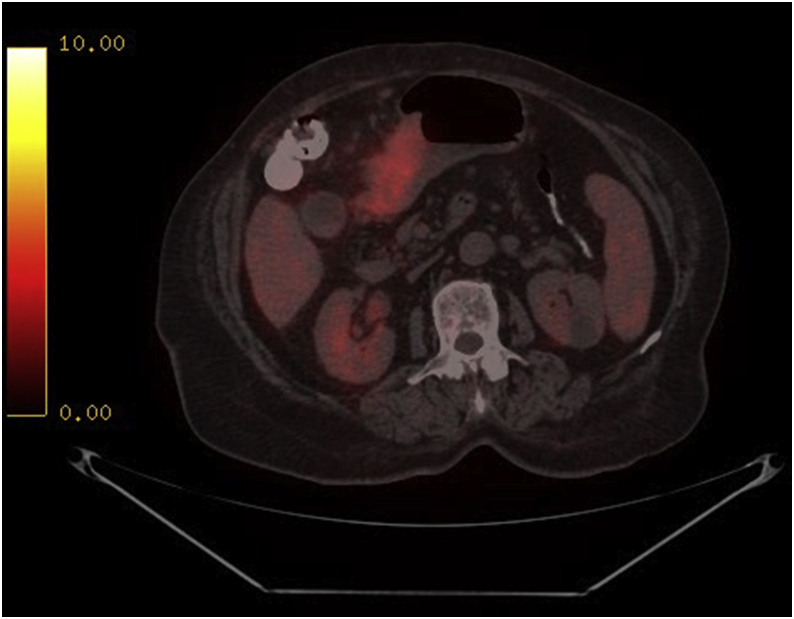
PET-CT scan pre-operatively showing focal FDG avidity in the antro-pyloric region suggestive of malignant mass.

On examination, she was hemodynamically stable. Abdominal examination revealed epigastric distension with no discrete lump or organomegaly. A contrast enhanced CT scan of the abdomen revealed a long 15 cm circumferential mural thickening of the terminal ileal loop, extending up to the site of the previous ileo-transverse anastomosis, with surrounding pericolic stranding. Upper GI endoscopy revealed a large ulcero-proliferative growth in the antro-pyloric region (Fig. 1). Pre-operative biopsy was suggestive of adenocarcinoma. Video-colonoscopy was grossly normal. PET scan was suggestive of an FDG avid lesion localised to the antro-pyloric region of the stomach with proximal dilatation of the gastric body and cardia (Fig. 2). The ileocolic anastomotic site did not reveal any increased nuclear uptake. The areas of thickening in the ileal loop were non FDG avid. There was no evidence of any FDG uptake in the liver or other solid organs.

At operation, she had an ulcero-proliferative growth in the antro-pyloric region causing luminal stenosis and gastric dilatation with enlarged lymph nodes in the surrounding perigastric region. A subtotal gastrectomy with resection of a mid ileal intraluminal polyp with excision of a suspicious omental nodule and anatomical repair of incisional hernia was done. Reconstruction was done by side-side hand sewn gastrojejunostomy using an isoperistaltic loop of jejunum. She developed left pleural effusion and respiratory distress, necessitating an ICU stay for 5 days. The patient was eventually discharged in a stable condition on day 10th of surgery. She received 5 cycles of Capecitabine and Bevacizumab based chemotherapy on an outpatient basis. Follow up CT abdomen performed in August 2017 was grossly normal, with no evidence of any recurrence or residual mass.

**Fig. 3:**
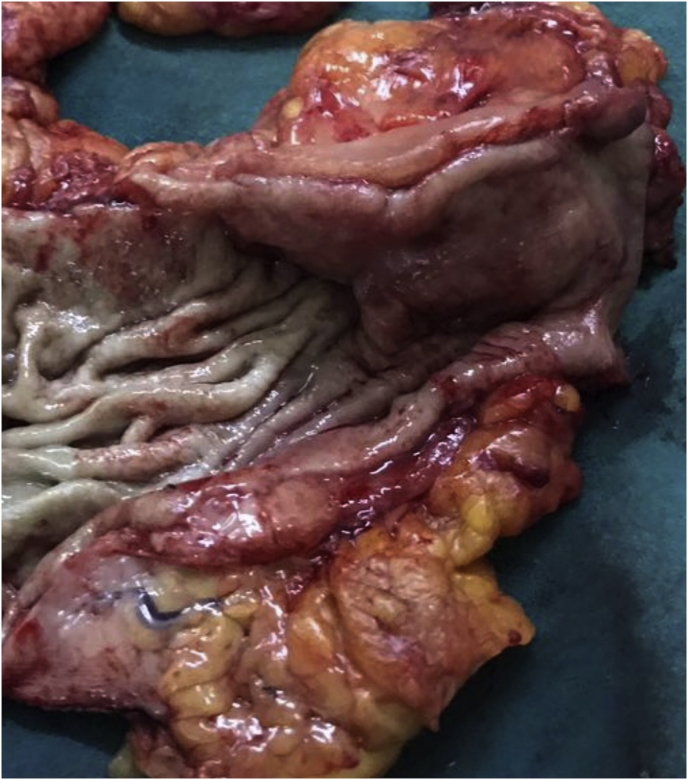
Gross specimen cut open along the longitudinal axis showing irregular proliferative growth in the antrum and pylorus. Note the normal overlying mucosa.

**Fig. 4:**
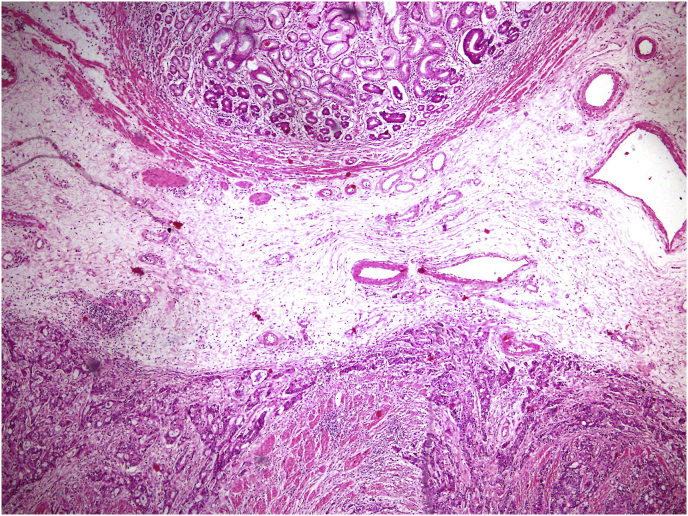
Histopathology (Hematoxylin and Eosin staining) showing proliferative glands with hyperchromasia in the submucosa and muscularis propria.

**Fig. 5:**
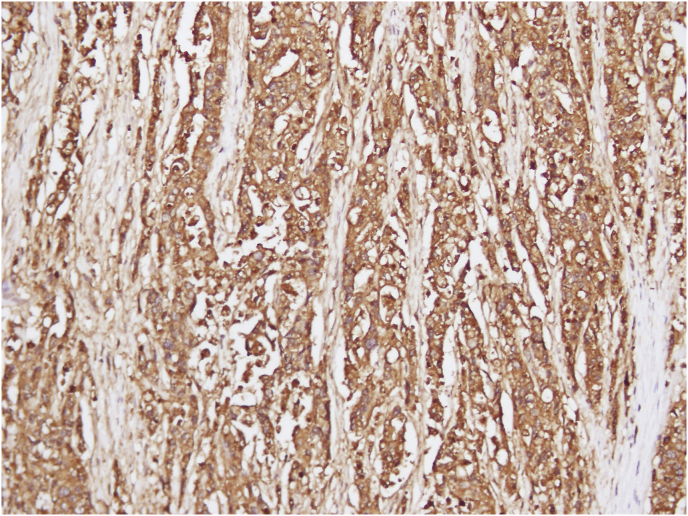
Immuno-histochemistry showing focal positivity for Carcino-embryonic antigen (CEA) suggestive of probable colonic origin.

**Fig. 6:**
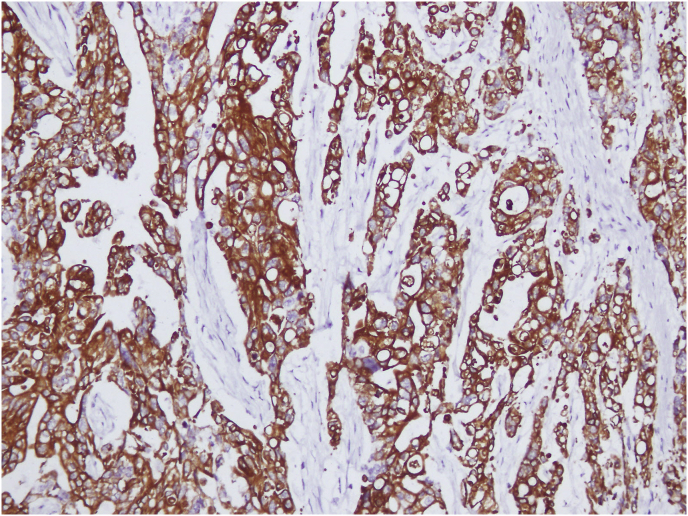
Immuno-histochemistry showing focal positivity for CK-20 (Cytokeratin) highly suggestive of colonic origin.

Gross examination of the specimen revealed a nodular ulcero-infiltrative mass on the mucosal surface of the stomach measuring 6 × 5.5 cm (Fig. 3). The overlying mucosa was intact. The tumor was infiltrating the muscle layer and extending into the greater omental fat. Microscopic examination showed a moderately differentiated adenocarcinoma, with lymphovascular invasion and perineural infiltration (Fig. 4). The resected margins and the omentum were free of tumor. Four out of 15 resected lymph nodes showed tumor metastases. On immunohistochemistry, the tumor was diffusely positive for CK 20 and CEA (Fig. 5), with focal positivity for CK 7 (Fig. 6). The resected ileal polyp and omental nodule were free of tumor. Based on the above findings, a diagnosis of moderately differentiated adenocarcinoma, intestinal type of colonic origin (pT3pN2pMx) was made.

## 3 Discussion

The published literature reveals a very low incidence of metastases to the stomach, with most studies reporting an incidence ranging between 0.2 and 0.7% [4]. In a series of 20 patients of gastric metastases from different primary origins, Campoli et al. reported a mean interval of 16 months (0–56 months) from the time of diagnosis of the primary neoplasm to the diagnosis of the gastric metastases [5]. In the majority of their patients, the diagnosis was established in less than a year. Clinically, most patients present with non-specific upper gastrointestinal symptoms like dyspepsia and bloating. In the series by Campoli et al., the most frequent symptom necessitating an endoscopy was gastric bleeding. Colonic metastasis to stomach are very rare, with few cases being described in the literature. Nujishima et al. reported a case of a distal gastric mass 1 year and 3 months following a left hemicolectomy for a transverse colon adenocarcinoma followed by a 6 month course of adjuvant Capecitabine and oxaliplatin based chemotherapy [6]. However, our patient remained asymptomatic till 6 years after undergoing the primary surgery for colonic malignancy followed by adjuvant chemotherapy. Upper GI contrast studies often reveal a characteristic single or multiple “bull's eye” lesion with a mucosal fold. The mass appears as a filling defect, with the overlying bridging mucosal fold representing the submucosal location of the mass. However, this is non-specific as many other pathological conditions may present with a similar finding such as lymphoma, spindle cell tumors, Kaposi's sarcoma, eosinophilic granuloma [7]. UGI endoscopy findings are variable, ranging from flat, plaque like lesions to ulcero-proliferative masses. There have been reports of diffuse infiltration (linitis plastica) as well with a primary lesion in the breast [8]. However, most authors have reported a predominance of ulcerated lesions. Most studies have reported single isolated lesions located in the distal body of the stomach (80%) [9].

The presence of metastasis to the stomach has been found to be a marker of advanced disease. Kobayashi et al., in their series of nine patients found concomitant distant metastases to lymph nodes and lungs in more than 50% of the cases [10]. Melanomas, owing to their greater tropism for the gastric submucosa, have a higher incidence of developing gastric metastases (22–25%) [11]. They tend to follow a more aggressive course than colonic or breast metastases. However, not much has been known about the course followed by colonic metastases to stomach. In the case reported by Nujishima et al., the patient developed widespread lymph nodal and peritoneal metastases 7 months after undergoing gastrectomy [6]. Our patient has been on regular out-patient follow up for 3 months and has had an uneventful post-operative course so far.

## 4 Conclusion

The present report describes a rare gastric tumor which turned out to be a metastasis from a caecal carcinoma for which the patient had undergone a right hemicolectomy as much as six years previously.

## Ethical approval

Patient consent had been obtained for possible publication of this case report.

## Source of funding

None.

## Author contribution

1. Samrat Ray: First and corresponding author; collected the data; first assistant in the surgery; prepared the first draft of the manuscript.
2. Amitabh Yadav: Chief operating surgeon. Supervised the manuscript preparation.
3. Sunila Jain: Histopathologist reporting the slides and doing immunohistochemistry. Inputs in the manuscript from the pathologist.
4. Samiran Nundy: The main supervisor of the project. Final corrections done by him.

## Conflicts of interest

None.

## Research registration number

NA.

## Guarantor

Samrat Ray.

## Patient consent

Patient informed consent had been obtained for possible publication of this case report.

## Provenance and peer review

Not commissioned, externally peer reviewed.

## Appendix A Supplementary data

SCARE Guideline Checklist SCARE Guideline Checklist
